# Biological Activities of Chinese Propolis and Brazilian Propolis on Streptozotocin-Induced Type 1 Diabetes Mellitus in Rats

**DOI:** 10.1093/ecam/neq025

**Published:** 2011-04-14

**Authors:** Wei Zhu, Minli Chen, Qiyang Shou, Yinghua Li, Fuliang Hu

**Affiliations:** ^1^College of Animal Sciences, Zhejiang University, Hangzhou 310029, China; ^2^Zhejiang Traditional Chinese Medicine University, Hangzhou 310053, China; ^3^Zhejiang Economic & Trade Polytechnic, Hangzhou 310018, China

## Abstract

Propolis is a bee-collected natural product and has been proven to have various bioactivities. This study tested the effects of Chinese propolis and Brazilian propolis on streptozotocin-induced type 1 diabetes mellitus in Sprague-Dawley rats. The results showed that Chinese propolis and Brazilian propolis significantly inhibited body weight loss and blood glucose increase in diabetic rats. In addition, Chinese propolis-treated rats showed an 8.4% reduction of glycated hemoglobin levels compared with untreated diabetic rats. Measurement of blood lipid metabolism showed dyslipidemia in diabetic rats and Chinese propolis helped to reduce total cholesterol level by 16.6%. Moreover, oxidative stress in blood, liver and kidney was improved to various degrees by both Chinese propolis and Brazilian propolis. An apparent reduction in levels of alanine transaminase, aspartate transaminase, blood urea nitrogen and urine microalbuminuria-excretion rate demonstrated the beneficial effects of propolis in hepatorenal function. All these results suggested that Chinese propolis and Brazilian propolis can alleviate symptoms of diabetes mellitus in rats and these effects may partially be due to their antioxidant ability.

## 1. Introduction

Propolis, a resinous substance collected from the buds of certain trees by bees, is a traditional herb medicine in many countries. More than 300 components have been found in propolis, mainly composed of phenolic compounds (e.g., flavonoids, aromatic compounds), terpenes and essential oil [1–3]. Propolis has been proven to have various bioactivities that are anti-pathogenic, immunoregulatory, antioxidative, anti-tumor, hepatoprotective and anti-inflammatory [3–5]. In China, propolis was authorized as a new material medicine and embodied in the Chinese Pharmacopeia in 2005 [6].

Diabetes mellitus leads to a series of complications such as retinopathy, neuropathy, kidney failure, heart disease and stroke. Therefore, diabetes mellitus can be a severe threat to public health and raises economic burden in the world [7]. It is estimated that the economic cost of diabetes came up to $174 billion in the USA in 2007 [8]. Due to the huge medical expenditure and complicated pathobiology of diabetes, research has focused on herbal medicine that might improve glucose control and lower the risk of complications [9–11].

Streptozotocin (STZ), a nitrosourea derivative isolated from *Streptomyces achromogenes*, can cause diabetes mellitus in rodents by selectively ruining pancreatic *β*-cells. STZ-induced diabetes in rodents is characterized by hyperglycemia, glucosuria, polyphagia, polydipsia, polyuria, body weight loss, hypoinsulinemia and hyperlipidemia [12, 13], and has been widely accepted as a model of type 1 diabetes mellitus for the studies of hyperglycemia and insulinopenia [14]. STZ is also used to develop a model of macro- or microvascular complications in diabetes [15, 16].

Our previous studies have shown that Chinese propolis helped to reduce fasting blood glucose (FBG) and improve oxidative stress and lipid metabolism in alloxan-induced diabetic rats [17]. In addition, clinic application of propolis benefited to control blood glucose [18]. A systematic study, therefore, was required to verify the antidiabetic effects of propolis and to reveal its possible mechanism. This study determined the effects of Chinese propolis and Brazilian propolis on STZ-induced type 1 diabetes in rats. The aims were to: (i) verify the antidiabetic effects of propolis; (ii) reveal its possible mechanism and (iii) compare the effects of Chinese propolis and Brazilian propolis.

## 2. Materials and Methods

### 2.1. Drugs and Reagents

Chinese propolis, produced by *Apis. mellifera* bees, was collected in Shandong Province, China, in 2007 and the main plant origin was the poplar (*Populus* sp.). Brazilian propolis (green or alecrim propolis), produced by *A. mellifera* bees, was obtained from the Hangzhou BEEWORDS Apiculture Co. Ltd. (Hangzhou, China). Polyethylene glycol (PEG) 400 and 6000 were purchased from the Shanghai Pudong Gaonang Chemical Factory (Shanghai, China); STZ was purchased from the ALEXIS Corporation (Switzerland). Liquid kits of glucose, total cholesterol (TC), triglyceride (TG), high-density lipoprotein cholesterol (HDL-C), low-density lipoprotein cholesterol (LDL-C), alanine transaminase (ALT), aspartate transaminase (AST), blood urea nitrogen (BUN), creatinine and urine albumin were purchased from DiaSys Diagnostic Systems (Shanghai) Co., Ltd, China. Test kits of superoxide dismutase (SOD), glutathione peroxidase (GSH-PX), malonaldehyde (MDA), nitric oxide (NO) and nitric synthetase (NOS) were obtained from Nanjing Jiancheng Biology Engineering Research Institute (Nanjing, China). NycoCard(r) HbA1c test kit was purchased from Axis-shield Poc AS (Norway).

### 2.2. Laboratory Animals

Male Sprague–Dawley (SD) rats weighing 200 ± 20 g were purchased from the Shanghai Laboratory Animal Center of the Chinese Academy of Sciences [Certificate No: SCXK(Shanghai) 2003-0003]. Afterwards, rats were reared at the Research Center of the Laboratory of Animal Science, Zhejiang College of Traditional Chinese Medicine (Hangzhou, China). The rats were acclimatized for 2 weeks prior to the experiment, during which time they were given free access to water and standard rat food. The animals were kept under a condition of physiological day/night rhythm, an ambient temperature of 23 ± 1°C, a humidity of 50–70% and noise <50 dB, which were in accordance with the Helsinki guidelines. The experimental protocol was approved by the Animal Ethics Committee of the Zhejiang University.

### 2.3. Induction of Diabetes Mellitus

Rats were fasted overnight before experiments. Fasted rats were injected intravenously through the tail vena with a single dose of 2% STZ (50 mg kg^–1^) dissolved in physiological saline solution (0.1 mmol citric acid/L). FBG and other biochemical indices were determined 7 days later. Thirty-two rats (mean weight: 270 ± 40 g) having an FBG concentration between 15 and 27 mmol L^–1^ were selected for the type 1 diabetes mellitus model. Diabetic rats were randomly divided into groups of model, Chinese, Brazilian and positive groups, with eight rats in each group. An additional eight rats without STZ induction were selected as the normal group.

### 2.4. Method of Drug Administration

Both Chinese and Brazilian propolis were extracted by 95% ethanol and then the two extracts were mixed with PEG 400 and PEG 6000 in a ratio of 1 : 1 : 1, respectively. The mixture was dissolved in physiological saline (10 mg propolis mL^–1^) for subsequent use. Physiological saline administered to normal, model and positive groups rats were also mixed with the corresponding concentration of PEG. These solutions were given to rats by oral intubation twice daily (09:00 and 15:00 h), continuously for 8 weeks. The dosage of each group is shown below.

Each rat in the Chinese group received intra-gastrically a dose of 10 mg Chinese propolis per 100 g body weight.

Each rat in the Brazilian group received intra-gastrically a dose of 10 mg Brazilian propolis per 100 g body weight.

Glucobay contains the active drug acarbose and helps to control blood sugar in diabetic patients. Glucobay was dissolved in physiological saline mixed with PEG at a concentration of 1 mg mL^–1^; each rat belonging to the positive group received intra-gastrically a dose of 1 mg glucobay per 100 g body weight.

Each rat belonging to the normal group and model group received intra-gastrically a dose of 1 mL physiological saline mixed with PEG per 100 g body weight.

### 2.5. Method of Measurement

The weight and FBG of all rats were measured weekly. Prior to sacrificing, rats were housed in metabolic cages for collecting 24-h urine to measure the urine biochemical indexes. Once rats were sacrificed, blood from the abdominal aorta was collected to measure the biochemical indexes. Hitachi 7020 entire automatic biochemistry analyzers (Hitachi Co. Ltd., Japan) were used to determine the content of TC, TG, HDL-C, LDL-C, BUN, serum creatinine (SCr), urine creatinine (UCr) and urine microalbumin. BT-815A semi-automatic biochemical analyzer (Shanghai Sanco Instrument Co. Ltd., China) was used to determine MDA, catalase (CAT), GSH-PX and SOD. glycated hemoglobin (HbA1c) was determined by the NycoCard Reader (Axis-Shield Company, Norway). Endogenous creatinine clearance rate (CCR) was calculated from the values of SCr, UCr and total 24-h urine volume. A 24-h urinary albumin-excretion rate (UAER) was calculated by urinary albumin concentration × 24-h urine volume (in mL). Kidneys and livers of dissected rats were removed and weighed. All the measurements were carried out in accordance with the instructions from manufacturers.

### 2.6. Data Analysis

Data were analyzed by the Statistical Package for the Social Sciences version 16 (SPSS16.0). One-way analysis of variance (ANOVA) test was performed and *post hoc* multiple comparisons were conducted with LSD. Results were presented as mean ± standard deviation (SD). A *P*-value < .05 was regarded as statistically significant.

## 3. Results

### 3.1. Inhibition on STZ-Induced Body-Weight Loss in Rats

Administration started 7 days following the injection of STZ and lasted 8 weeks. All rats were weighed after 10 h of fasting overnight before treatment (Week 0) and every week during treatment.

**Figure 1:**
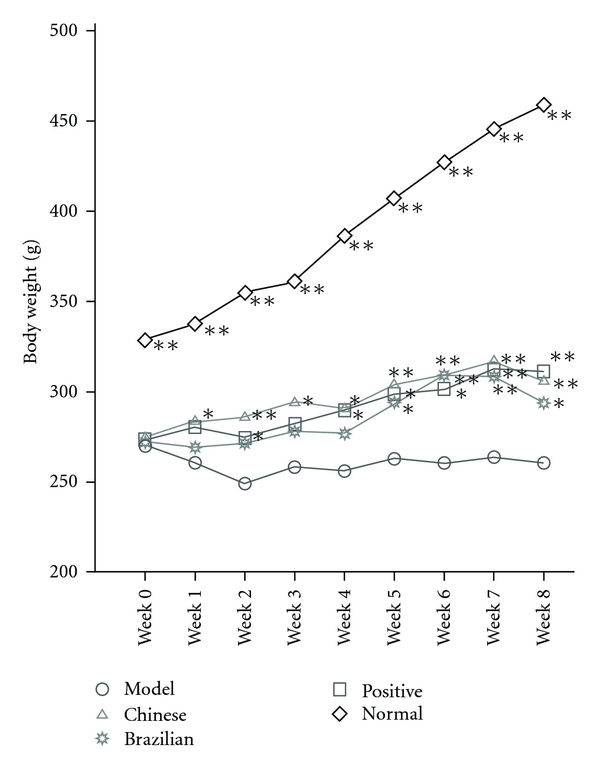
Inhibition on body-weight loss. Values represent the means ± SD, *n* = 8. *P < .05 and **P < .01, compared with the model group. Model group and normal group rats: received physiological saline; Chinese group rats: received Chinese propolis; Brazilian group rats: received Brazilian propolis and Positive group rats: received glucobay.

As shown in Figure 1, the body weight of STZ-treated rats was significantly lower than that of the normal rats without STZ treatment over the experimental period (*P* < .01), indicating the induction of diabetes by STZ. The body weight of all propolis-treated rats increased at various levels. The most interesting thing was that Chinese propolis-treated rats (Chinese group) exhibited a significantly elevated body weight during the whole period of treatment (*P* < .01 or *P* < .05) compared with the rats in the model group (*P* < .01 or *P* < .05). Moreover, the Brazilian propolis-treated rats (Brazilian group) showed a significantly elevated body weight from the 5th week to the 8th week (*P* < .01 or *P* < .05), compared with the rats in the model group in the same period. Thus, both Chinese propolis and Brazilian propolis were considered to prevent body-weight loss induced by STZ in diabetic rats.

### 3.2. Improvement of Blood Glucose Level of Diabetic Rats

**Figure 2:**
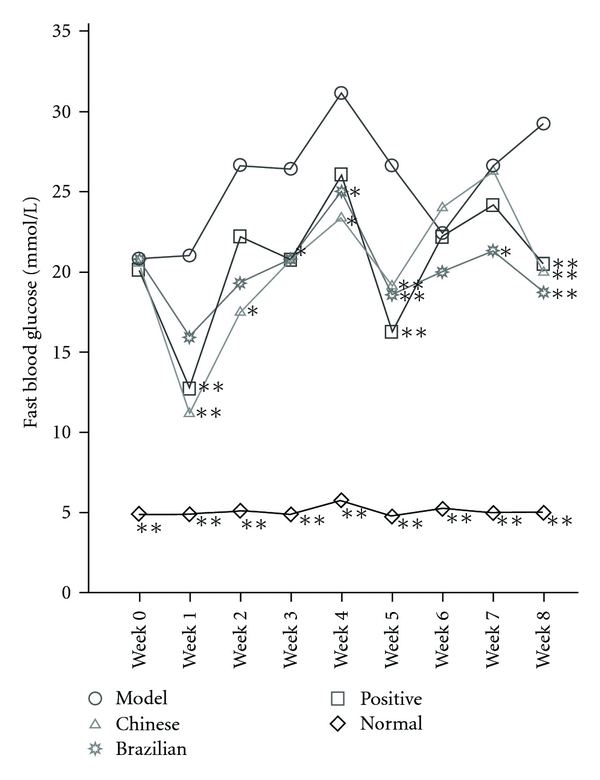
Reduction of fasting blood glucose level. Values represent the means ± SD, *n* = 8. *P < .05 and **P < .01, compared with the model group. Model group and normal group rats: received physiological saline; Chinese group rats: received Chinese propolis; Brazilian group rats: received Brazilian propolis and Positive group rats: received glucobay.

**Figure 3:**
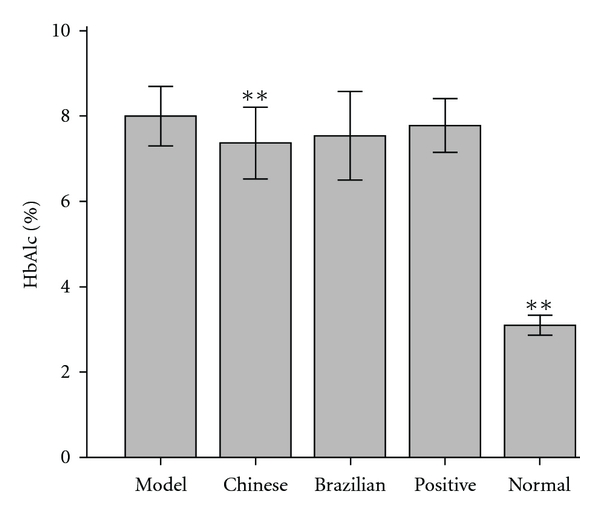
Changes in serum HbAlc level. Each column represents the means ± SD, *n* = 8. **P < .01, compared with the model group. HbAlc, glycosylated hemoglobin.

All rats were fasted for 10 h overnight followed by FBG-level measurement during the experiment. HbAlc levels in rats were measured before sacrifice. As shown in Figures 2 and 3, FBG and HbAlc levels in model group rats were significantly elevated compared with normal rats (*P* < .01). From Weeks 1 to 5 and at Week 8, FBG levels in Chinese group rats decreased significantly compared with those in the model group rats (*P* < .05 or *P* < .01). From Weeks 3 to 5 and from the 7th to the 8th week, FBG levels in the Brazilian group rats were evidently lower than those in model group rats (*P* < .05 or *P* < .01). At Week 6, propolis showed no improvement on FBG levels in diabetic rats. As shown in Figure 3, the HbAlc level was significantly reduced from 8 ± 0.35% in model group rats to 7.33 ± 0.41% in Chinese group rats (*P* < .01).

### 3.3. Restoration of STZ-Damaged Hepatorenal Function

Diabetes mellitus can damage tissues and organs seriously. Therefore, the protective effect of propolis on the kidneys and renal function was measured. In our study, the levels of kidney weight (KW), kidney weight/body weight (KW/BW), SCr, BUN, CCR, and UAER were used to evaluate the degree of renal damage.  Table 1 showed that BUN, UAER, CCR, KW and KW/BW levels in healthy rats were clearly lower in the normal group rats than those in the model group rats (*P* < .01), whereas SCR levels did not differ significantly between diabetic rats and normal rats. Compared with the model group rats, BUN levels in the Brazilian group were significantly suppressed (*P* < .01); KW/BW levels in the Chinese group were obviously inhibited (*P* < .05); propolis-treated rats had a similar UAER, which was obviously reduced (*P* < .05). 

Liver weight (LW), liver weight/body weight (LW/BW), ALT and AST were used to estimate the degree of hepatic damage.  Table 2 shows that the levels of AST, ALT and LW/BW in healthy rats were clearly lower in the normal group rats than those in the model group rats (*P* < .01), whereas LW/BW levels showed no marked difference between model rats and normal rats. Compared with model rats, LW levels in Chinese and Brazilian groups significantly increased (*P* < .01 and *P* < .05, resp.); levels of AST and ALT in Chinese and Brazilian groups decreased significantly (*P* < .05 and *P* < .01, resp.).

### 3.4. Suppression of Serum Oxidative Stress

Free radical plays an important role in the onset of diabetes mellitus and its complications.  Table 3 shows that both Chinese propolis and Brazilian propolis ameliorated blood oxidative stress in diabetic rats at various degrees. Chinese propolis, but not Brazilian propolis, significantly inhibited the MDA level (*P* < .01). In contrast, Brazilian propolis, but not Chinese propolis, obviously reduced the NOS level and increased the SOD level (*P* < .01). The GSH-Px level did not change significantly in diabetic rats compared with normal rats. Neither propolis nor positive drug had any effect on the levels of NO and CAT. 

### 3.5. Protection against Hepatorenal Oxidative Stress

Results of the effect of propolis on hepatorenal oxidative stress are presented in Table 4. Increased MDA levels in liver and kidney indicated an exasperated oxidative stress in diabetic rats. Liver antioxidase activity in model group rats was lower than that in normal group rats, as evidenced by the decreased level of SOD activity (*P* < .05). Chinese propolis showed no improvement on MDA level and antioxidase activity in liver, whereas Brazilian propolis had an apparent improved effect on liver MDA and SOD level (*P* < .05 and *P* < .01, resp.). No significant differences in liver CAT level were detected between model rats and normal rats, but liver CAT levels in Brazilian group rats visibly increased compared with model rats (*P* < .05). Renal MDA levels in Chinese group and Brazilian group rats significantly decreased (*P* < .05 and *P* < .01, resp.), compared with those in model group rats. Renal CAT and SOD levels in model rats were lower than those in normal rats (*P* > .05), whereas renal GSH-Px levels in model group rats significantly increased compared with normal rats (*P* < .05). Although renal SOD and CAT levels did not differ obviously between model group rats and normal group rats, both Brazilian and Chinese propolis obviously increased the CAT level (*P* < .05).

### 3.6. Amelioration of Blood Lipid Metabolism

Disturbance of lipid metabolism emerges in diabetic rats.  Table 5 showed that levels of LDL-C, HDL-C, and TC did not change noticeably in model group rats, whereas TG level was greatly increased compared with that in normal group rats (*P* < .05). Propolis treatment showed no obvious reduction in TG level, but TC level in Chinese group rats was decreased by 16.6% (*P* < .05).

## 4. Discussion

Hyperglycemia is regarded as one of the main causes of diabetes complications, and the improvement of glycemic control reduces the incidence of complications [19–21]. HbAlc is an ideal indicator for long-term glycemic control and the risk of diabetic complications. A 1% increase in the HbA1c level is accompanied by a significant increase in incidence of cardiovascular events, whereas a decrease in the HbAlc level to 6.5% is accompanied by a reduction of 10% in the risk of macro- and microvascular diseases [22, 23]. Water extracts of Brazilian propolis and an active constituent caffeoylquinic acid suppressed postprandial blood glucose rise in SD rats by inhibiting maltase activities [24]. Our previous works showed that ethanol extracts and water extracts of Chinese propolis helped reduce levels of FBG and HbAlc in alloxan-induced diabetic rats but had no effect on postprandial blood glucose in healthy mice [25]. In this study, Chinese and Brazilian propolis suppressed the increase of blood glucose levels and weight loss in diabetic rats. Compared with model group rats, an 8.4% reduction of the HbAlc level in Chinese propolis-treated rats (*P* < .01) further confirmed the hypoglycemic effect of Chinese propolis. These results suggest that Chinese and Brazilian propolis may prevent the progression of diabetes mellitus by a different action pathway.

Diabetes can damage hepatorenal function and lead to diabetic nephropathy (DN). It is estimated that DN affects 15–25% of type 1 diabetes patients and 30–40% of type 2 diabetes patients [26]. Microalbumin (from a 24-h urine collection), SCr, CCR and BUN are important prognostic markers for kidney disease and are useful measurements of glomerular filtration rate, whereas ALT and AST are commonly used for screening liver problems. Brazilian propolis showed an obvious inhibitory effect on BUN level and both Chinese and Brazilian propolis decreased the UAER, ALT and AST levels. These results demonstrated the protection effect of propolis on hepatorenal function in diabetic rats. This conclusion was consistent with several previous reports, which showed that propolis and its active constituent caffeic acid phenethyl ester (CAPE) have apparent therapeutic effects on liver and kidney lesions in animal models [27–30].

**Figure 4:**
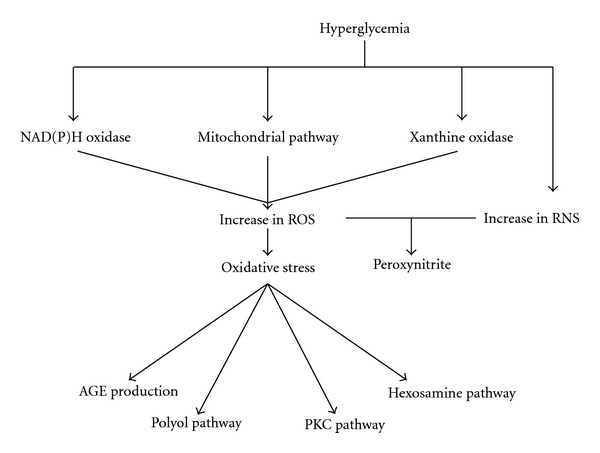
ROS as a common upstream event of diabetes. ROS are believed to be an underlying mechanism of diabetes. Hyperglycemia induced oxidative stress by stimulating mitochondrial pathway, NAD(P)H oxidase and xanthine oxidase, and subsequently activated various pathways including PKC pathway, hexosamine pathway, polyol pathway, production of AGE and so on. In addition, hyperglycemia disregulated the expression of reactive nitrogen species (RNS) which reacts with superoxide forming peroxynitrite, a highly reactive oxidant. In this experiment, Chinese and Brazilian propolis were certificated to inhibit the increase of MDA and improve the antioxidase activity in STZ-damaged rats. In addition, Brazilian propolis decreased serum NOS level, which suggested that the improvement of propolis on diabetes may partially be attributed to the inhibition of oxidative stress.

Multifold pathways, including increased polyol pathway flux, increased hexosamine pathway, production of advanced glycation end product (AGE) and protein kinase C (PKC) activation, are involved in diabetic complications. Increased reactive oxygen species (ROS), induced by hyperglycemia-activated electron-transport chain in mitochondria mainly, are believed to be an underlying mechanism linking all of these factors (Figure 4) [31, 32]. Over-expression of SOD or uncoupling proteins (UCPs) may block the mitochondrial electron-transport chain and result in a reduction of ROS and deactivation of these pathways [31]. Clinical trials also proved that improvement of oxidative stress may prevent the progression of both type 1 and type 2 diabetes [33, 34]. Propolis has a strong antioxidative activity and is confirmed to inhibit increase of MDA level and improve antioxidase activity in the animal model and patients [35–37]. Previous experiments reported that propolis can prevent tissue damage from oxidative stress by decreasing the overproduction of MDA and superoxide anion and by restoring respiratory control ration in mitochondrial tissue [38, 39]. In this experiment, all results suggested that the protective effect of propolis on hepatorenal function is partially attributed to antioxidant activity and it may act by affecting the mitochondrial respiratory chain. In addition, we found that not all antioxidase levels in diabetic rats were decreased, and a clearly increased kidney GSH level in model rats was detected, which is identical to some other reports [36, 37]. Based on the results in Tables 3 and 4, we concluded that Brazilian propolis had a more comprehensive antioxidant effect than Chinese propolis.

NO, synthesized by various NOS, is an important messenger molecule and brings both beneficial and deteriorating effects on the human body. Excessive NO reacts with superoxide to form a strong oxidant peroxynitrite (Figure 4), which is linked to various diseases including diabetes [40]. Normally, type 1 and type 2 diabetes patients are associated with dysfunction of the endothelial cell, which decreases the bioavailability of NO and leads to vasoconstriction and hypertension [41]. Scavenging of ROS by antioxidants helps to enhance NO bioavailability and alleviate tissue damage [42]. However, mediated by inflammatory factor and other cytokines, increased NO levels are found in diabetic patients and in the animal model [42, 43]. Overproduction of NO promotes the progression of diabetic retinopathy and early-stage DN; hence the inhibition of increased NO or NOS may prevent progression [42, 43]. In this experiment, we found a higher level of NO and NOS in model rats compared with normal rats. Propolis has an obvious anti-inflammatory effect and inhibits the increase of NOS in the animal model [44]. Brazilian propolis, as an inhibitor of NOS through an anti-inflammatory effect, may decrease the level of NO.

Type 1 and type 2 diabetes present a different dyslipidemia: Type 1 diabetes is usually characterized by normal levels of LDL-C and HDL-C and an increased TG level, whereas type 2 diabetes is associated with a reduced HDL-C level, an elevated TG level and a normal LDL-C level [45]. Hyperlipidemia leads to atherosclerosis and chronic cardiovascular disease (CVD). Reduction in serum cholesterol level reduces the risk of CVD substantially [45, 46]. Our previous study found that Chinese propolis reduced levels of TC, LDL-C and very low-density lipoprotein-cholesterol (VLDL-C) in diabetic rats [18]. In this experiment, the manifestation of dyslipidemia in STZ-induced diabetic rats was similar to type 1 diabetes, as Chinese propolis also significantly decreased TC level. LDL oxidation plays an important role in atherosclerosis and propolis inhibits lipid peroxidation *in vitro* and *in vivo* [47–49]. These results suggested that propolis may reduce the incidence of CVD by preventing the increase of cholesterol level and LDL oxidation.

Activities and chemical composition of propolis often varied with plant source, extraction, collecting time and collecting bee [50–52]. *Baccharis dracunculifolia* DC is the main plant source of Brazilian propolis, and prenylated *p*-coumaric acids are the predominant biologically active substances in this propolis [52]. Chinese propolis is a poplar-type propolis, and flavonoids, cinnamic acids and their esters are the main active components in this propolis [53]. The difference in antidiabetic effect between Chinese and Brazilian propolis may be due to the chemical differences.

In conclusion, this study demonstrated that Chinese and Brazilian propolis significantly prevent the progression of STZ-induced diabetes in SD rats and alleviate increased oxidative stress in diabetic rats. Chinese propolis can improve lipid metabolism in rats. Further studies are necessary to reveal whether the anti-inflammatory properties of propolis are involved in the antidiabetic effect.

## Funding

Chinese Ministry of Agriculture grant (Project number: NYCYTX-43) and Zhejiang Provincial Natural Science Foundation of China grant (Project number: R3090332).

## Figures and Tables

**Table 1:** Protection against STZ-induced renal damage.

Group	Model	Chinese	Brazilian	Positive	Normal
KW (g)	3.10 ± 0.30	3.31 ± 0.17	3.24 ± 0.22	3.18 ± 0.43	2.54 ± 0.21**
KW/BW (‰)	11.91 ± 0.72	10.74 ± 0.67*	11.24 ± 0.76	10.38 ± 1.55**	5.68 ± 0.43**
BUN (mmol L^–1^)	14.19 ± 2.41	12.66 ± 3.59	9.23 ± 1.89**	10.50 ± 3.02**	4.14 ± 0.70**
SCr (umol L^–1^)	57.75 ± 3.33	56.25 ± 4.23	56.25 ± 3.41	59.25 ± 3.96	57.25 ± 5.42
UAER (mg/24 h)	0.45 ± 0.21	0.27 ± 0.16*	0.26 ± 0.18*	0.19 ± 0.13**	0.12 ± 0.05**
CCR (mL min^–1^)	2.02 ± 0.53	1.97 ± 0.25	1.97 ± 0.29	1.08 ± 0.54**	0.81 ± 0.43**

Values represent the mean ± SD, *n* = 8. KW, kidney weight; BW, body weight; BUN, blood urea nitrogen; SCr, serum creatinine; UAER, urinary albumin-excretion rate; CCR, creatinine clearance rate.

*P < .05 and **P < .01, compared with the model group.

**Table 2:** Prevention of STZ-induced hepatic damage.

Group	Model	Chinese	Brazilian	Positive	Normal
LW (g)	11.61 ± 0.73	13.30 ± 0.68**	12.73 ± 1.04*	13.40 ± 1.03**	12.09 ± 1.24
LW/BW (‰)	44.94 ± 5.02	43.22 ± 2.70	44.12 ± 2.81	43.63 ± 6.80	27.01 ± 1.68**
ALT (IU L^–1^)	128.67 ± 51.06	93.00 ± 18.75*	85.83 ± 21.78**	123.83 ± 34.61	45.83 ± 5.91**
AST (IU L^–1^)	250.17 ± 65.67	195.50 ± 23.74*	172.50 ± 13.28**	226.67 ± 64.85	127.67 ± 7.99**

Values represent the mean ± SD, *n* = 8. LW, liver weight; ALT, alanine transaminase; AST, aspartate transaminase.

*P < .05 and **P < .01, compared with the model group.

**Table 3:** Suppression of blood oxidative stress.

Group	Model	Chinese	Brazilian	Positive	Normal
NO (*μ*mol L^–1^)	19.40 ± 9.04	14.70 ± 2.96	16.26 ± 2.69	19.13 ± 5.60	10.41 ± 1.60**
NOS (U mL^–1^)	46.93 ± 4.83	46.98 ± 3.21	36.47 ± 6.38**	36.97 ± 3.35**	35.30 ± 6.04**
SOD (U mL^–1^)	39.42 ± 14.30	44.46 ± 11.66	54.53 ± 3.41**	57.16 ± 4.07**	53.24 ± 5.51**
CAT (U mL^–1^)	9.65 ± 0.83	9.97 ± 1.04	11.04 ± 1.07	9.60 ± 1.11	12.69 ± 2.11**
GSH-Px (*μ*mol L^–1^)	687.88 ± 48.29	682.35 ± 48.89	663.38 ± 80.87	674.76 ± 44.08	692.22 ± 35.85
MDA (nmol L^–1^)	5.15 ± 0.55	3.61 ± 0.80**	4.80 ± 2.11	2.50 ± 0.59**	3.27 ± 0.41**

Values represent the mean ± SD, *n* = 8. NO, nitric oxide; NOS, nitric synthetase; SOD, superoxide dismutase; CAT, catalase; GSH-px, glutathione peroxidase; MDA, malonaldehyde.

*P < .05 and **P < .01, compared with the model group.

**Table 4:** Reversal of hepatorenal oxidative stress.

Group	Model	Chinese	Brazilian	Positive	Normal
Liver SOD (U mL^–1^)	69.33 ± 6.90	81.38 ± 16.79	91.65 ± 17.62**	76.43 ± 9.32	86.56 ± 13.57*
Liver CAT (U mL^–1^)	83.28 ± 6.28	94.57 ± 19.22	97.95 ± 19.47	87.24 ± 10.24	97.32 ± 12.54
Liver GSH (*μ*mol L^–1^)	265.18 ± 27.86	338.09 ± 26.48	358.99 ± 146.43*	306.55 ± 68.18	314.78 ± 46.19
Liver MDA (nmol L^–1^)	2.67 ± 0.66	2.11 ± 0.49	1.84 ± 1.10*	1.84 ± 0.76*	1.82 ± 0.52*
Renal SOD (U mL^–1^)	80.34 ± 2.42	81.44 ± 12.34	84.09 ± 7.04	92.44 ± 16.93	84.99 ± 3.78
Renal CAT (U mL^–1^)	30.69 ± 2.48	37.49 ± 3.17*	37.27 ± 2.18*	42.73 ± 9.91**	34.80 ± 4.48
Renal GSH (*μ*mol L^–1^)	729.80 ± 70.47	660.75 ± 113.02	653.66 ± 253.09	639.71 ± 124.49	554.75 ± 45.22*
Renal MDA (nmol L^–1^)	2.83 ± 0.68	2.06 ± 0.28*	1.85 ± 0.29**	2.16 ± 0.42*	1.97 ± 0.31**

Values represent the mean ± SD, *n* = 8.

*P < .05 and **P < .01, compared with the model group.

**Table 5:** Regulation of blood lipid metabolism (mmol L^−1^).

Group	Model	Chinese	Brazilian	Positive	Normal
LDL-C	0.36 ± 0.02	0.30 ± 0.12	0.32 ± 0.05	0.30 ± 0.09	0.34 ± 0.05
HDL-C	0.96 ± 0.21	0.83 ± 0.12	0.84 ± 0.17	0.92 ± 0.17	0.97 ± 0.16
TG	1.90 ± 0.47	1.75 ± 0.65	1.62 ± 0.44	1.91 ± 0.86	1.11 ± 0.27*
TC	1.86 ± 0.33	1.57 ± 0.22*	1.66 ± 0.24	1.75 ± 0.27	1.71 ± 0.21

Values represent the mean ± SD, *n* = 8. LDL-C, low-density lipoprotein-cholestereol; HDL-C, high-density lipoprotein-cholestereol; TG, triglyceride; TC, total cholesterol.

*P < .05 and **P < .01, compared with the model control group.
